# A review of perioperative anesthesia and analgesia for infants: updates and trends to watch

**DOI:** 10.12688/f1000research.10272.1

**Published:** 2017-02-08

**Authors:** Lizabeth D Martin, Nathalia Jimenez, Anne M Lynn

**Affiliations:** 1 University of Washington School of Medicine, Department of Anesthesiology & Pain Medicine, Seattle Children’s Hospital, Seattle, WA, USA

**Keywords:** perioperative anesthesia and analgesia, neonatal and infant pharmacology, pharmacokinetics, pharmacodynamics

## Abstract

This review focuses on pharmacokinetics and pharmacodynamics of opioid and non-opioid analgesics in neonates and infants. The unique physiology of this population differs from that of adults and impacts drug handling. Morphine and remifentanil are described as examples of older versus recently developed opiates to compare and contrast pharmacokinetics and pharmacodynamics in infants. Exploration of genetics affecting both pharmacokinetics and pharmacodynamics of opiates is an area of active research, as is the investigation of a new class of mu-opiate-binding agents which seem selective for analgesic pathways while having less activity in pathways linked to side effects. The kinetics of acetaminophen and of ketorolac as examples of parenteral non-steroidal analgesics in infants are also discussed. The growth in regional anesthesia for peri-operative analgesia in infants can fill an important role minimizing intra-operative anesthetic exposure to opioids and transitioning to post-operative care. Use of multi-modal techniques is recommended to decrease undesirable opiate-related side effects in this vulnerable population.

## Introduction

This article begins by highlighting the features of neonatal and infant physiology that differ from those of adults and impact drug handling. Definitions of pharmacokinetic terms and a brief introduction to models for drug metabolism will be presented. Morphine and remifentanil will be used as examples of older versus recently developed opiates to compare and contrast pharmacokinetics in infants. The pharmacodynamics of these agents is important to consider in this vulnerable group. Opioid-related side effects include respiratory depression, tolerance, ileus, urinary retention and pruritus. Genetics affecting both pharmacokinetics and pharmacodynamics of opiates has begun to be explored. The growth in regional anesthesia for peri-operative analgesia in infants can fill an important role, minimizing intra-operative anesthetic exposure and transitioning to post-operative care. Use of multi-modal techniques to decrease opiate-related side effects in vulnerable infants is also desirable. The kinetics of acetaminophen and of ketorolac (as examples of parenteral non-steroidal analgesics) in infants will be reviewed. Investigation of a new class of mu-opiate-binding agents, which seem selective for analgesic pathways while having less activity in pathways linked to side effects is a new area with clinical studies beginning in adults; this constitutes an important area to follow.

Clearly, the information to be presented will be our selection from the literature and cannot be a comprehensive review of all literature of these drugs or drug classes. References to some of the authors’ own work is used for convenience and knowledge of study performance details, not to suggest that other work is not equally important.

## Physiology

The physiology of the neonate and infant differs in many aspects from that of the adult; some of these differences are important factors for drug handling. Total body water is a higher percentage of body weight in infants, reaching adult values by the age of 8 to 10 years ^1^. Liver and kidney function is not fully developed at birth, affecting handling of many drugs. The maturation of organ function occurs over several months during the first year of life. Drug development in the past 10 to 20 years has focused on agents whose metabolism is less dependent on normal renal or liver function (or both) since aging adults often have compromise in the function of these organs. This is beneficial for infants who also have immature function. Remifentanil is one obvious example of such a drug.

Hepatic enzymes, including both the P450 system and the glucuronidation pathways, are immature at birth. Maturation occurs over the first few months of postnatal life, at different rates for different P450 variants. The kidney is important for eliminating drugs or their metabolites. Drugs that are metabolized by glucuronidation (to increase solubility for excretion) will have delayed removal in the first months of life. Sulfation then becomes more important as a solubility pathway for urinary excretion. In infants, glomerular filtration rates start at approximately 10% of adult normal values, reaching these by 12 months of age. Renal tubular function also matures over the first 6 months. This immature function can result in the accumulation of metabolites and is particularly problematic when those metabolites have active effects.

## Pharmacokinetics

Pharmacokinetics, clearance (Cl), volume of distribution (Vd), and elimination half-life (t ½) are common pharmacologic terms, and familiarity can be helpful when reviewing this literature. Pharmacokinetics is defined as the study of drug disposition by patients; it is affected by absorption (important for non-intravenous [non-IV] routes of administration), distribution, metabolism, and elimination. Familiarity with common pharmacologic terms can be helpful when reviewing this literature ^2^ and are briefly defined below since most readers are familiar with them.

Cl describes the removal of drug from a volume of plasma per unit of time (mL/min, L/hour or normalized as mL/min per kg, L/hour per 70 kg).

Vd, another volume term, describes the apparent volume necessary to account for all drug if it were present at the same concentration as measured in plasma or serum (L/70 kg or mL/kg). Importantly, Vd is a theoretical, rather than physiologic, volume. Its usefulness is to show how widely distributed a drug is in the body.

The t ½ is the time needed to change blood concentration by 50% (minutes, hours). In 4 to 5 half-lives, 94% to 97% of drug is removed, respectively. Half-life is affected by both Cl and Vd. Drugs with a higher Cl and smaller Vd have a shorter duration of action and are more titratable, often desirable in anesthesia practice. As novel anesthetic drugs with these features have been developed, the term context-sensitive half-time has come into use. This term refers to the time to change drug concentration by 50% (that is, half-life) in the context of the duration of administration. Context-sensitive half-time pharmacokinetic models for IV anesthetic drugs have been described ^2, 3^. Many newer agents have been developed to achieve small context-sensitive half-times which are unchanged regardless of the duration of drug infusion.

Limitations on blood sampling volumes for pediatric patients, particularly significant in infants, have encouraged the development of computer-generated pharmacokinetic mathematical modeling schemes which use sparse blood concentration data from pediatric patients. Population-based models exist which use factors such as age and weight as regular covariates and introduce additional elements to improve the fit of the model in concentration versus time graphs. Weight-based allometric models incorporate weight by using exponential factors (0.75 for Cl and 1 for Vd) based on enzyme function characteristics. These models consider age-appropriate factors such as creatinine or bilirubin to improve fit. Both models have been used to study morphine and remifentanil handling in infants ^4– 8^.

### Genetics influencing pharmacokinetics

An active and rapidly evolving area of research is the study of genetic factors influencing pharmacokinetics and pharmacodynamics of analgesics. Although most studies involve animal models and the effect of genetic variants is sometimes difficult to extrapolate directly into clinical practice, there are examples of important metabolic-genetic variants pertinent to pediatric populations ^9^. A prime example is codeine, a pro-drug that is entirely dependent on its metabolism for its analgesic effect. Its active metabolite, morphine, is produced by O-demethylation by the CYP2D6 (Cytochrome P450 2D6) enzyme, which is highly polymorphic. Differences in CYP2D6 alleles can result in significant differences in opiate effect because of variable CYP2D6 enzyme activity levels. Patients range from poor to ultra-rapid metabolizers on the basis of their combination of alleles. In clinical practice, patients who are poor metabolizers will not achieve any analgesia with codeine use, whereas patients who are ultra-rapid metabolizers have a higher risk of respiratory depression ^10– 12^. Similar concerns have been reported with tramadol, hydrocodone, and oxycodone, resulting in the publication of dosing guidelines considering CYP2D6 genotypes ^11, 13, 14^.

The frequency of genetic polymorphisms varies between racial and ethnic populations. According to the previous example, European Caucasians are more likely to carry normal function CYP2D6 alleles compared with Asians and African-Americans ^15^. East Africans (Ethiopians), on the other hand, are more likely to exhibit duplications of the CYP2D6-gene (indicative of ultra-rapid metabolism), which predisposes them to serious adverse reactions ^16^. Studying ethnic, racial, and genetic factors in opiate metabolism remains important in helping us individualize care for patients of all ages.

### Morphine

**Figure 1.**
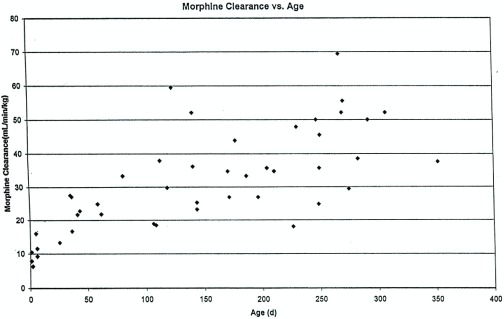
Morphine clearance (mL/min per kg) versus age in infants receiving morphine. Reprinted with permission from Wolters Kluwer Health ^23^.

Studies of morphine pharmacokinetics have been reported by several groups ^17, 18^. The focus here will be on the author’s work (AL), which is known in detail. In 1987, a small study in 10 infants showed that they had pharmacokinetic parameters different from those reported in adults, and a lower Cl and large Vd resulted in a prolonged t ½ ^19^. Study of a larger cohort of 49 infants and toddlers, all receiving morphine by IV infusion after cardiac surgery, showed the same pattern; adult values for Cl were reached by the age of 6 months ^20^. A subsequent study of 26 infants who received morphine by IV infusion after non-cardiac surgeries found Cl in non-cardiac infants to be significantly greater than in infants after cardiac surgery, and non-cardiac surgical infants reached adult morphine Cl values by 1 to 3 months of age ^21^. In all of these studies, inter-patient morphine Cl variability was high, giving values two- to three-fold different for same-aged infants, a problem seen with most “older” opiates ^17, 22, 23^ ( Figure 1). This variability makes it difficult to predict the duration of both desired and undesired morphine effects in any individual infant.

Klimas and Mikus recently suggested that active morphine metabolite morphine-6-glucuronide (M-6-G) was responsible for 85% to 96% of morphine analgesia based upon a literature review of published studies reporting these concentrations in adults ^24^. Bouwmeester *et al.* have reported M-6-G concentrations in infants in several reports but linking these concentrations or M-6-G/morphine ratios specifically to analgesia has not been analyzed ^17, 25^. Neonates and young infants have immature glucuronidation enzymes, and the amount of M-6-G that is produced in this population is unclear. The role of genetic polymorphisms in UDT-transferase glucuronidation also plays a role in inter-individual variability and is incompletely studied.

## Pharmacodynamics

Pharmacodynamics refers to the effects of the drug on the patient; these may be therapeutic or undesired (adverse) ^2^. For most clinicians, pharmacodynamics drives our administration of drugs. Pharmacodynamics are affected by many factors; for opiates, these include differences in receptor morphology (OPRM gene variant SNP 118 A/G), differences in target site concentrations of drug (ABCB 1/MDR 1 transporter), or effects downstream from drug reaching receptors and binding, such as differences in catechol-O-methyltransferase (COMT) metabolism of catechols (472 G>A SNP). Studies of these genetic differences in opiate effects in different populations have been increasing and should continue to be an active area of research ^26– 29^. Genetic influences on pain perception also affect the pharmacodynamics of pain medications ^30, 31^.

Respiratory effects of opiates have been one of the factors limiting their use in infants. Studies linking drug concentrations to effects are few. In 1993, a study of morphine concentrations at steady state in 30 infants receiving IV morphine infusions after cardiac surgery found that a plasma morphine concentration of less than 20 ng/mL was associated with hypercarbia in 15% versus 67% in those with morphine concentrations over 20 ng/mL, independent of age. This suggested that morphine infusions set to target a plasma concentration of 20 ng/mL could minimize the risk of respiratory depression in infants ^32^. Unfortunately, the inter-patient variability in morphine pharmacokinetics makes predicting morphine concentration for an individual infant more difficult (see above discussion, Figure 1).

In 2000, a study of 83 infants demonstrated superior analgesia with morphine infusions compared with intermittent bolus dosing (high pain scores in 13% versus 32%) ^23^. No difference in continuous oximetry readings of less than 90% in room air or time to oral intake was found. However, carbon dioxide (CO _2_) response curve slopes did show evidence of ventilatory depression in 4 (7%) out of 57 infusion-group infants.

A suggested course to facilitate extubation at surgery end is to use 0.05 mg/kg morphine as a loading dose, then infusion at 5 to 10 μg/kg per hour in neonates, and 10 to 20 μg/kg per hour in infants, adjusting to each individual infant’s response.

### Remifentanil

The unpredictable pharmacokinetics and potential for undesired respiratory depression with morphine have led to the investigation of opioids with Cl that is less reliant on hepatic or renal function. Remifentanil is a mu-agonist, metabolized by tissue esterases, so its pharmacokinetics is unaffected by changes in hepatic or renal function. Important for use in infants, these tissue esterases are functional at birth, so age-related changes in pharmacokinetics have not been seen. Onset and offset are rapid, so IV infusion is the most common administration route. The context-sensitive half-time is reported as 3 to 5 minutes, independent of duration of infusion ^33^. Davis *et al.* did a multi-center trial in 60 infants undergoing pyloromyotomy, comparing remifentanil infusion at 0.55 ± 0.2 μg/kg per minute to halothane 0.4%, with field block with bupivacaine for post-operative analgesia in both groups. Although there was no difference in time to extubation, no infant in the remifentanil group had any new abnormality on post-operative pneumograms, where three patients in the volatile-only group did ^34^. Crawford *et al.* demonstrated that remifentanil can provide excellent conditions for orotracheal intubation in infants without the use of muscle relaxants. Twenty-four infants, pretreated with glycopyrrolate 10 μg/kg, were randomly assigned to propofol/succinylcholine or propofol/remifentanil 3 μg/kg, and intubating conditions were similar with no adverse events ^35^. A subsequent study including 64 children who were 0 to 3 years old, stratified in three age groups (0 to 3 months, 4 to 12 months, and 1 to 3 years) ^36^, suggested that the age-specific bolus dose of remifentanil (ED50) to facilitate tracheal intubation is slightly higher (3.7 μg/kg) among the 4 to 12-month-old group. In this study, all patients were also pretreated with glycopyrrolate.

Undesired effects, including “chest wall rigidity” and bradycardia, have been reported, as can be seen with other fentanyl derivatives, and can lead to difficulty with ventilation and cardiovascular compromise. More importantly for anesthetic use, remifentanil analgesia is gone within 15 to 30 minutes of stopping infusions. Some authors have suggested that tachyphylaxis occurs quickly and that remifentanil use leads to higher opiate requirements; however, this has not been reported in infants.

Remifentanil avoids the challenges of pharmacokinetic variability that are seen with morphine; however, rapid offset can pose a challenge for post-operative analgesia. If post-operative pain can be approached with regional techniques, remifentanil seems a workable choice to allow early extubation after infant surgery.

## Regional anesthesia in infants

Potential deleterious effects of anesthetic toxicity in the developing brain, as well as heightened awareness of negative long-term sequelae of exposure to painful stimuli early in life, have been recently highlighted ^37– 40^. Regional anesthesia techniques, including local anesthetic infiltration, neuraxial blockade, and selective peripheral nerve blocks, have the potential benefit of decreasing anesthetic exposure during surgery as well as optimizing pain management in the post-operative period ^41, 42^. Decreasing opioid use while maintaining analgesia is particularly desirable in neonates and infants.

Regional techniques are technically challenging in newborns and infants and may require additional expertise. Assessment of risks versus benefits of regional anesthesia in infants is ongoing. Several large prospective multi-center studies, including the UK epidural audit ^43^ and the Pediatric Regional Anesthesia Network (PRAN) multi-center report ^44^, demonstrate that regional anesthesia in children is safe, although neonates and infants are a small subset of the reported populations ^45^. Despite the extremely low reported serious complication rates in all of these studies, case reports of permanent neurologic injury, including paralysis, exist in the literature ^46^. Careful risk/benefit analysis, meticulous technique, and experience are recommended, especially prior to placing high lumbar or thoracic epidurals, where damage to the spinal cord can lead to devastating complications.

Over the past 15 years, improved equipment designed specifically for infants and neonates, increased utilization and quality of ultrasound technology, and emerging studies investigating the pharmacokinetics of local anesthetics in neonates have improved the safety and feasibility of regional anesthesia in this age group. Neonates and young infants are at a higher risk of local anesthetic toxicity because of immature enzyme systems and lower plasma protein levels ^45^, which develop unpredictably with gestational age as well as vary from individual to individual ^47^. Recent pharmacokinetic studies of epidural and peripheral local anesthetic in this age group contribute to the development of safe dosing guidelines. In 2012, Calder *et al.* studied 31 infants age 40 to 63 weeks post-menstrual age (post-menstrual age is the time between the first day of last menstrual period and birth [gestational age] plus the time elapsed after birth [chronological age]) having single-injection or continuous epidural for hernia repairs and demonstrated safe plasma ropivacaine and bupivacaine levels without accumulation after doses of 1.5 mg/kg followed 2 hours later by 0.2 mg/kg per hour infusions ^48^. Similarly, for single-injection peripheral nerve blocks, Suresh *et al.* demonstrated low plasma bupivacaine levels after single-shot transversus abdominis plane (TAP) blocks with 0.125% bupivacaine 1 mL/kg in neonates ^49^.

## Neuraxial

Caudal block, which is most commonly employed for urologic, orthopedic, and general pediatric surgical procedures, provides post-operative pain relief; duration of the block depends on the local anesthetic type, volume, and adjuvants that are used ^50– 52^. Concern has been raised about neuraxial epinephrine and risk of spinal cord ischemia ^53^. Epinephrine use should be reserved for initial test dosing with single-dose caudal or epidural neuraxial catheters and should be avoided as an additive in infusions. Opioids prolong the duration and may improve the quality of the block ^54^, but the use as an adjuvant has been controversial because of associated side effects, including respiratory depression, nausea, pruritus, and urinary retention ^54, 55^. Whether the benefits of caudal epidural opioids outweigh the risks is actively debated ^56, 57^. Minimizing opioid-related side effects post-operatively in infants and neonates may be best achieved by starting with opioid-free epidural infusions and supplementing with intermittent IV opioids if needed.

Clonidine has been shown to prolong the duration of epidural blocks ^58, 59^, although young infants seem particularly sensitive to sedating side effects and apnea may occur. For this reason, clonidine is not used as an adjuvant in infants of less than 10 kg at our institution. Safety data for neuraxial use of ketamine, midazolam, and neostigmine remain insufficient to support their use as epidural adjuvants ^56^.

Use of spinal block in pediatric patients remains limited and is used mainly as an awake technique for ex-premature infants at risk for post-operative apnea ^60^. The duration of spinal anesthesia in children is less than 60 to 75 minutes and this requires close coordination between anesthesia and surgery as well as short surgical times ^61^. However, spinal anesthesia provides a reliable, dense block and is useful for surgeries on the lower body in select patients.

## Peripheral nerve blocks

Most well-described peripheral nerve blocks can be performed on neonates and infants. Precision is mandatory and meticulous attention must be given to the weight-based dosing limitation of local anesthetics. Axillary or femoral blocks can be used to facilitate peripherally inserted central catheter (PICC) line placement or treat limb ischemia ^62, 63^, illioinguinal or TAP blocks can be performed for trunk surgery ^49^, and paravertebral blocks for thoracotomy ^64^. Infants and neonates tend to have superficial, well-defined anatomy and tissue planes which can be visualized with high-frequency ultrasound. Excellent needle visualization is required to safely perform ultrasound-guided peripheral nerve blocks in this population.

## Interesting new trends in opiate development

Another area of active research is the development of “biased ligands” of the mu-opioid receptor (MOR). Biased ligands are molecules that can discriminatively engage signaling pathways of a receptor ^65^, offering the advantage of separating desirable (analgesic) from undesirable (respiratory depression, constipation, nausea) opiate effects. Research suggests that, for the MOR, opioid-induced analgesia is due to signaling of G protein-coupling inhibition of nociception but that side effects are modulated by signaling of beta-arrestin ^66, 67^. Novel biased ligands have been developed based on the concept of selective signaling. TRV130, a novel MOR G protein-biased ligand, has shown potent analgesic effect with minimal gastrointestinal dysfunction and respiratory suppression compared with morphine in rodents ^68^. In humans, a phase III clinical trial in adult male volunteers reported that low doses of TRV130 (1.5 and 3 mg) were well tolerated, demonstrating a better analgesic response with less reduction of respiratory drive and nausea when compared with 10 mg of morphine ^69^. Higher doses of TRV130 (4 mg) also produced less respiratory depression than morphine but were associated with increased reports of nausea and headache. Another molecule recently identified, PZM21, also shows selective activation of the G-protein pathway with a better side effect profile compared with TRV130 in rodents ^70^. However, human studies are not yet available. Development of opioid-biased ligands is an exciting area of new drug development that has not reached the point of pediatric investigation but that suggests a theoretical alternative for opioid analgesic treatment for infants with a better analgesic-to-adverse effect ratio.

Much recent attention in adults and adolescents has concerned the problem of long-term use of opiates and best practices to minimize this risk ^71^. We believe this is an important issue but needs to be balanced by concerns of long-term effects of undertreating pain in infants.

## Non-opiate analgesics

Depending on the patient and procedure, other categories of analgesics may be considered to complement or replace opioids. Table 1 summarizes the analgesics described in this article. Description of the pharmacokinetics/dynamics in infants of acetaminophen (focusing on IV route) and of ketorolac (the only widely available parenteral non-steroidal anti-inflammatory drug [NSAID] in the US) may be of particular benefit in infants as they do not work via opiate receptors and do not have effects on respiration or consciousness.

**Table 1.** Pharmacokinetic and clinical considerations of common analgesic medications in infants.

Medication	Metabolism	Metabolite	Renal excretion	Pharmacokinetic considerations	Clinical considerations
Morphine	Hepatic	Yes, active	Yes	Inter-individual variability	Titrate to effect, small doses of 0.05 mg/kg
Remifentanil	Plasma esterase	No	No	Predictable duration	No long-acting analgesia
Ketorolac	Hepatic	Yes, isomers different	Yes	R-isomer accumulation	Single dose only for infants < 6 months old. For patients > 6 months old, limit Q6 hour dosing to 72 hours total duration.
Acetaminophen	Hepatic	Yes, toxic	No	Intravenous formulation	Every-6-hour dosing interval preferred

### Ketorolac

Ketorolac tromethamine is available in oral and parenteral forms. As the only widely available IV NSAID in the US, it has been used as an analgesic adjunct and has been shown to decrease opioid requirements in post-operative patients. It works by inhibiting the cyclooxygenase system which decreases prostaglandin synthesis and the inflammatory cascade. It does not affect consciousness or respiration but does have effects on gastric mucosa, renal perfusion, and platelet function. Baseline renal or platelet dysfunction is a relative contraindication.

A prospective randomized trial of 70 infants and children showed no difference in chest tube drainage or bleeding complications and no difference in median change in creatinine after congenital cardiac surgery with or without 48 hours of ketorolac therapy post-operatively ^72^. Aldrink *et al.* reported a retrospective review of 57 infants who received multiple doses of ketorolac (mean of eight doses every 6 hours) post-operatively. Ten had a bleeding event (three required transfusion). Risk factors identified were age of less than 21 days and gestational age of less than 37 weeks ^73^.

To further explore the pharmacokinetic, safety, and respiratory effects in infants, we did a single-center randomized, blinded, placebo-controlled study in infants 6 to 18 months admitted after surgery. The kinetics analyses were done for stereo-specific isomers since animal studies suggested that the S-isomer was a more potent analgesic ^74^ and studies in children suggested differences in isomer handling ^75, 76^. Thirty-seven infants were enrolled, 23 following craniectomy surgery. All received morphine for breakthrough pain. A marked difference in isomer Cl was found, with the S-isomer cleared fourfold faster than the R-isomer (4.4 ± 2.8 mL/min per kg versus 1.0 ± 0.6, respectively). The t ½ was 64 ± 24 minutes (S) versus 198 ± 77 minutes (R). Serum concentrations of the S-isomer fell below adult “therapeutic” values (as reported by Stanski *et al.*) by 4 hours. Modeling of doses of 0.5 mg/kg or 1 mg/kg showed complete Cl of the S-isomer by 4 hours, but accumulation of the R-isomer with each dose. As a single-dose study, no adverse effects on renal function (creatinine and urine volume), gastric mucosa (guaiac testing), or platelet function (surgical drain amount in craniectomy babies) were observed. Continuous oximetry showed no episodes of desaturation in either placebo or ketorolac groups. Unanswered is the possibility that toxicity may relate to the R-isomer concentrations which rise with repeat dosing ^77^. In 2- to 6-month-old infants, when the same protocol was used, similar results were found, and the S-isomer cleared fivefold faster than the R-isomer of ketorolac. The t ½ was 33 minutes (S) versus 191 minutes (R) ^78^. Safety data are limited in neonates or infants at higher risk for renal dysfunction except in the studies by Gupta *et al.* and Aldrink *et al.* detailed above, and caution is advised in this patient population. In our institution, we administer ketorolac 0.5 mg/kg every 6 to 8 hours for a maximum of 72 hours to infants older than 6 months and a single dose 0.5 mg/kg for infants 1 to 6 months. Although the retrospective review by Aldrink *et al.* in a 0- to 3-month-old age group suggests that scheduled dosing of ketorolac may be well tolerated in those over 3 weeks of age or over 37 weeks’ gestational age, pharmacokinetic and prospective safety data for this practice are based on small samples.

### Acetaminophen

Acetaminophen (or paracetamol in European or UK literature) is the most widely used analgesic/antipyretic administered in oral or rectal forms. In 2002, propacetamol, an IV pro-drug, was developed, and in 2009 an IV active form of acetaminophen became available in Europe. In the US, IV acetaminophen was approved by the US Food and Drug Administration more recently, so many of the reports on its use come from European or UK sites. One advantage of this drug is its long history of use in the oral form, meaning that safety information in children and infants is available. Hepatic toxicity from overdose of acetaminophen and accumulation of the metabolite N-acetyl-p-benzoquinone imine (NAPQI) is very uncommon in infants because they have immature function of P450 enzyme, specifically CYP2E1, and make much lower concentrations of this metabolite. The ideal analgesic concentration has been incompletely studied, but Anderson *et al.* reported that acetaminophen concentrations of 10 mg/L resulted in analgesia in children post-tonsillectomy ^79^. This concentration is commonly targeted as therapeutic but whether it applies in all circumstances is unexplored.

In 2005, Anderson *et al.* reported a population pharmacokinetic study of IV propacetamol in children. The authors included 846 acetaminophen concentrations from seven previous studies in a total of 144 children. The bioavailability was 0.5, Cl was 16 L/hour per 70 kg, increasing from 1.27 L/hour per 70 kg in premature infants (gestation 27 weeks) to 84% of adult values by age 1 year. Vd (peripheral) decreased from 45 L/70 kg in prematures (27 weeks) to adult values by the age of 6 months. The authors predicted that dosing of propacetamol of 30mg/kg (15mg/kg paracetamol) every 6 hours would result in acetaminophen concentrations of 10 mg/L in pediatric patients ^80^.

Allegaert *et al.* (2004) reported kinetics of a single dose of IV acetaminophen in 30 neonates showing lower Cl in infants of less than 37 weeks’ gestation (8.1 L/hour per 70 kg) compared with those of 37 to 41 weeks’ gestation (11.9 L/hour per 70 kg), noting marked inter-patient variability. The t ½ was prolonged at 277 minutes in the premature group compared with 172 minutes in the term infants, and again large variability was noted ^81^. Population pharmacokinetics of acetaminophen in infants was reported by Allegaert *et al.* in 2011. They included 158 neonates (58 prematurely born) from four studies with 943 acetaminophen concentration. Cl from this larger group of infants was 5 L/hour per 70 kg, and adult Cl of 16.2 L/hour per 70 kg was found at the age of 1 year. The authors’ suggestion was use of one loading dose of 20 mg/kg followed by 10 mg/kg every 6 hours to target an acetaminophen concentration of 11 mg/L in infants with a gestational age of 32 to 44 weeks ^82^.

The safety profile and kinetics of IV acetaminophen were studied by Palmer *et al.* in 50 neonates given repeated doses post-operatively ^83^. Cl was 5.2 L/hour per 70 kg, with a large Vd of 76 L/70 kg. Elevated bilirubin was correlated with decreased Cl. Daily hepatic enzyme levels remained normal in 49 of the 50 infants, increasing in one after five doses and recovering when acetaminophen was stopped. The authors suggest 15 mg/kg dosing every 6 hours in term infants and a reduction for hyperbilirubinemia ^83^.

Ceelie *et al.* did a blinded randomized controlled trial in 71 infants, born after 37 weeks gestation up to 1 year of age, treated after major abdominal or thoracic surgery. Infants received IV acetaminophen 30 mg/kg per day in four doses or morphine IV infusions at 4 to 16 μg/kg per hour after a standardized anesthetic with one dose of morphine 30 minutes before surgery end. Rescue for pain was with morphine bolus dosing in both groups. Pain scores and the number needing rescue were the same in both groups. Total morphine was lower in the acetaminophen group (122 μg/kg over 48 hours versus 357 μg/kg per 48 hours); naloxone was given to three in the morphine group and none in the acetaminophen group ( *P* = not significant) ^84^.

## Conclusions

The clinical application of pharmacokinetic/pharmacodynamics of opiates (morphine or remifentanil) or NSAIDs (ketorolac or IV acetaminophen) to our care of infants who require surgery has room for much further study. Further investigation of biased ligand compounds which may be able to separate analgesia (G-protein activation) from side effects (beta-arrestin recruitment) may offer distinct advantages in infant peri-operative care. Until then, it seems reasonable to consider multi-modal therapy to minimize undesirable opiate side effects. Variability of Cl of older opiates such as morphine supports using small doses (0.05 mg/kg morphine) and titrating for effect. Remifentanil avoids the variability issue but requires infusion to maintain effect, and analgesia is gone quickly (<30 minutes) once it is discontinued, which may not be appropriate for the post-operative infant. Regional analgesic techniques can help bridge intra-operative to post-operative analgesia but require practitioners skilled in its use in these smallest patients.

Ketorolac in a single-dose study did not show adverse effects on hepatic, gastrointestinal, or renal function but raises the concern of accumulation of the R-isomer with repeated doses and its unknown relation to toxicity. Small retrospective studies suggest caution in ketorolac use in prematures (born at <37 weeks) or in young neonates (<3 weeks). For infants, acetaminophen does have IV kinetic information available to guide dosing and therefore can be a part of a multi-modal analgesic regime in most infants. Non-pharmacologic adjuncts which we have not presented in this forum, including use of oral glucose, non-nutritive sucking, and swaddling, should also be considered.
